# Differences in weight status among Australian children and adolescents from priority populations: a longitudinal study

**DOI:** 10.1038/s41366-024-01471-0

**Published:** 2024-02-02

**Authors:** Thomas Lung, Anagha Killedar, Sarah Taki, Li Ming Wen, Michelle Dickson, Kirsten Howard, Louise Baur, Patrick Kelly, Simone Sherriff, Alison Hayes

**Affiliations:** 1 Sydney School of Public Health, Faculty of Medicine and Health, University of Sydney, Sydney, NSW Australia; 2 The George Institute for Global Health, University of New South Wales, Sydney, NSW Australia; 3 Menzies Centre for Health Policy and Economics, University of Sydney, Sydney, NSW Australia; 4 Health Promotion Unit, Population Health Research & Evaluation Hub, Sydney Local Health District, Sydney, NSW Australia; 5 NHMRC Centre of Research Excellence in the Early Prevention of Obesity in Childhood (EPOCH), University of Sydney, Sydney, Australia; 6 Poche Centre for Indigenous Health, Faculty of Medicine and Health, University of Sydney, Sydney, NSW Australia; 7 Specialty of Child and Adolescent Health, Sydney Medical School, University of Sydney, Sydney, NSW Australia

**Keywords:** Obesity, Epidemiology, Risk factors

## Abstract

**Background and significance:**

Australia has a high level of cultural and linguistic diversity, including Aboriginal and Torres Strait Islander peoples. Children from specific cultural and ethnic groups may be at greater risk of overweight and obesity and may bear the additional risk of socioeconomic disadvantage. Our aim was to identify differences in body-mass index z-score (zBMI) by: (1) Cultural and ethnic groups and; (2) Socioeconomic position (SEP), during childhood and adolescence.

**Subjects/Methods:**

We used data from the Longitudinal Study of Australian children (*n* = 9417) aged 2–19 years with 50870 longitudinal measurements of zBMI. Children were classified into 9 cultural and ethnic groups, based on parent and child’s country of birth and language spoken at home. These were: (1) English-speaking countries; (2) Middle East & North Africa; (3) East & South-East Asia; (4) South & Central Asia; (5) Europe; (6) Sub-Saharan Africa; (7) Americas; (8) Oceania. A further group (9) was defined as Aboriginal and Torres Strait Islander from self-reported demographic information.

Longitudinal cohort analyses in which exposures were cultural and ethnic group and family socioeconomic position, and the outcome was zBMI estimated using multilevel mixed linear regression models. We stratified our analyses over three periods of child development: early childhood (2–5 years); middle childhood (6–11 years); and adolescence (12–19 years).

**Results:**

Across all three periods of child development, children from the Middle East and North Africa, the Americas and Oceania were associated with higher zBMI and children from the two Asian groups were associated with lower zBMI, when compared to the referent group (English). zBMI was socioeconomically patterned, with increasingly higher zBMI associated with more socioeconomic disadvantage.

**Conclusions:**

Our findings identified key population groups at higher risk of overweight and obesity in childhood and adolescence. Prevention efforts should prioritize these groups to avoid exacerbating inequalities in healthy weight in childhood.

## Background

The high prevalence of overweight and obesity among children and adolescents remains a global public health issue, but there is evidence that overweight and obesity rates have plateaued in many advanced economies, including Australia [1]. However, inequalities exist in the population distribution of childhood overweight and obesity [2], with higher prevalence among children from: culturally and linguistically diverse (CALD); Aboriginal and Torres Strait Islander; and socioeconomically disadvantaged families [3]. Consistent with Australia’s National Obesity Strategy 2022–32, we use the term “priority populations” to encompass these three groups as they tend to have a higher prevalence of overweight or obesity in childhood [4]. There is evidence childhood overweight and obesity rates in some of these priority populations continued to rise between 1997 and 2015 [5]. However, further evidence is required to identify which cultural and ethnic groups are priority populations with excess weight in childhood and adolescence.

This is pertinent, as nearly half of all Australians were born overseas or have parents who were born overseas [6] and high rates of immigration [7] continue to increase the cultural and linguistic diversity of Australia. There is a complex interrelationship between cultural, socioeconomic, and environmental risk factors that may influence the adiposity of children differently from various backgrounds at different stages of child development and schooling. These factors include cultural and social norms towards weight status and levels of physical activity [8]; lifestyle behaviours [9, 10]; genetic variability, transitions from traditional to Western diets, length of residency and level of acculturation [11].

Cross-sectional studies have found higher odds of overweight and obesity for some priority populations, including immigrants from low-and-middle-income countries compared to children from high-income countries and Aboriginal and Torres Strait Islander children [5, 12–14]. Waters et al. describe the “double disadvantage” of ethnicity and socioeconomic position (SEP), with respect to overweight or obesity; this has been corroborated by analysis of cross-sectional school surveys [15] and national longitudinal data from Australia [16]. However, no studies have examined the relationship between priority populations and body-mass index z-score (zBMI) from early childhood (2 years) to the end of adolescence (19 years) and during which stage of childhood differences in zBMI [17] arise.

Families from priority populations often face challenges accessing early childhood health services or health interventions for various reasons including lack of knowledge of the existing services, language barriers and cultural differences [18]. Most services, programs and research addressing obesity prevention are focused primarily for English-speaking populations which could widen and exacerbate existing inequalities in childhood obesity. To reduce these inequities, there is a need to understand which priority groups confer a higher risk of obesity at different periods of childhood to ensure interventions supporting healthy growth are culturally relevant and accessible to diverse populations.

The purpose of this study was to identify the priority populations with higher zBMI by child developmental age, using Australian longitudinal cohort data on children aged 2–19 years between 2006 and 2018.

## Methods

### Study population

We used data from the “baby” (B) and “kindergarten” (K) cohorts from the Longitudinal Study of Australian Children (LSAC). The LSAC is a population-based, nationally representative longitudinal study of child development which used a two-stage clustered sampling technique identifying eligible children from the Medicare Australia enrolment database [19]. Further details of the sampling design and study methodology are described elsewhere [19].

Data collection began in 2004 and was collected biennially in waves. The initial B cohort comprised 5,107 children aged between 0 and 1 years of age and the K cohort was comprised of 4983 children aged between 4 and 5 years of age. In this study, we used data from waves 2–8 for both the B and K cohorts, representing 9417 children aged between 2 and 19 years of age.

#### Ethics declarations

Ethics approval for this study was received from the University of Sydney Human Research Ethics Committee (Project Number 2022/699) and the Aboriginal Health & Medical Research Council (Application ID: 35578980). The Australian Institute of Family Studies Ethics Committee approved each wave of the LSAC and use of the LSAC dataset was in accordance with the terms in the deed of licence.

### Data collection

Informed written consent was obtained and trained interviewers who conducted face-to-face or audio computer assisted interviews and anthropometric measurements with parents and children.

### Outcome

The outcome variable of interest in this study was zBMI. Anthropometric measurements of the children’s height and weight were taken at each wave by a trained interviewer. Weight was measured to the nearest 50 g using: HoMedics digital BMI bathroom scales in waves 2 and 3; and Tanita body fat scales in waves 4–8. Height was measured using an Invicta (Waves 2–3) and laser (Waves 4–8) stadiometer. BMI was calculated using height and weight measurements and transformed to age- and sex-adjusted zBMI according to the World Health Organization (WHO) growth standards for ages up to 5 [20] and WHO growth reference for children aged 5–19 years [21]. zBMI scores were calculated using the WHO’s SAS macro package [20] and values that were less than -5 and greater than 5 were excluded due to the biological implausibility of those values [20].

### Exposures—Priority populations

**Fig. 1:**
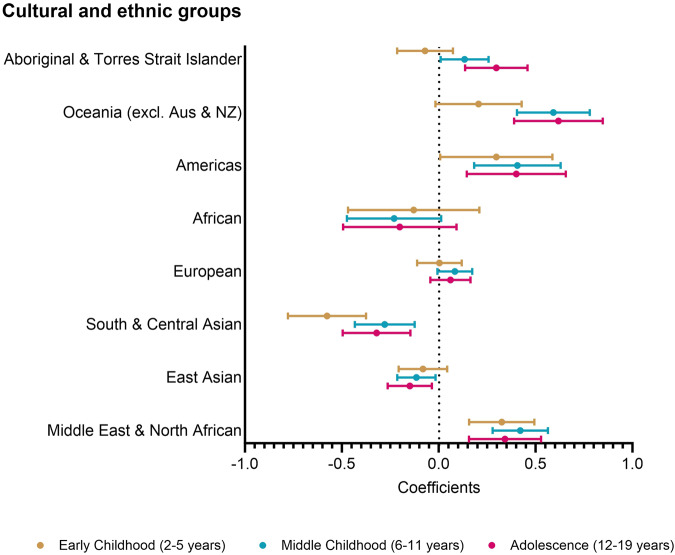
Association of zBMI with cultural and ethnic groups during early childhood, middle childhood and adolescence. Multilevel mixed linear regression models plotting association of zBMI with cultural and ethnic groups, with the English-speaking group as the referent. Aus: Australia, NZ: New Zealand.

**Fig. 2:**
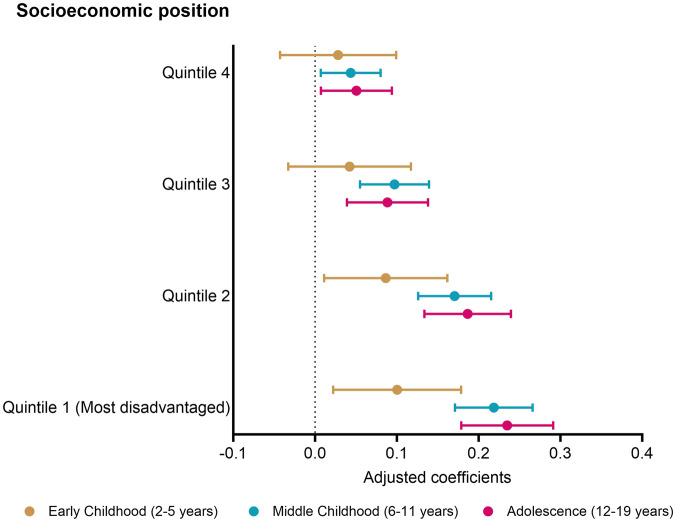
Association of zBMI with socioeconomic position during early childhood, middle childhood and adolescence. Multilevel mixed linear regression models plotting association of zBMI with socioeconomic position quintiles, with the most advantaged (Quintile 5) as the referent.

We divide priority populations into two exposures: (1) Cultural and ethnic groups; (2) Socioeconomic position. Cultural and ethnic groups were defined using the Australian Bureau of Statistics’ Standard Classification of Countries [22] and languages [23] into nine distinct groups: (1) English-speaking countries; (2) Middle-East and North Africa; (3) East and South-East Asia; (4) South and Central Asia; (5) Europe; (6) Sub-Saharan Africa; (7) Americas; (8) Oceania excluding Australia and New Zealand; and (9) Aboriginal and Torres Strait Islander peoples. A combination of variables captured in the LSAC based on a core set of cultural and language indicators set by the ABS Standards for Statistics on Cultural and Language Diversity was used [24]. These were ‘*country of birth’* for the child, primary and secondary parent; *‘main language spoken at home’* for the child (K cohort only), primary and secondary parent; and ‘*Aboriginal and Torres Strait Islander status*’. In the absence of information on self-identification of cultural group, children were classified using a sequence of decision rules based on the seven variables and accounting for cultural norms and historical migration patterns where possible. Further details of how children were classified, and a list of countries and languages spoken for each group are provided in the Supplementary Information (Supplementary Tables 1, 2 and Figs. 1, 2).

The second exposure was SEP, defined in LSAC combining data on parents’ education, occupation and family income, which was then converted into a z-score and categorised into quintiles [25].

### Statistical analysis

We separated the analyses on three defined periods of child development [26, 27]: early childhood (2–5 years), middle childhood (6–11 years) and adolescence (12–19 years). Waves 2 and 3 of the B cohort were used to analyse early childhood and both cohorts were pooled to analyse middle childhood (B cohort waves 4–6, K cohort waves 2–4) and adolescence (B cohort waves 7–8, K cohort waves 5–8). Given the similarity in recruitment of children, sampling strategies and distribution of values for zBMI and priority populations (see Supplementary Tables 3, 4), we believe this pooling approach to be reasonable. Descriptive statistics at baseline were used to characterise the study population.

We used multilevel linear regression to model the association of priority populations on zBMI during the three periods of childhood. To account for the repeated measurement for each variable, all models consisted of the same two-level hierarchical structure, the measure of zBMI at each time point (level 1), nested within individuals (level 2). For analyses where cultural and ethnic group was the exposure, an unadjusted model was fitted. When SEP was the exposure, we adjusted for cultural and ethnic group as a confounder. Adjusted regression coefficients (β), 95% confidence intervals (CIs) and intraclass correlation coefficients (ICC) were reported.

Survey weights were provided separately for the B and K cohorts in the LSAC, however we did not apply the weights in our analysis as we pooled the B and K cohort for most of our analyses, meaning weighting would not have been possible. Sex-stratified analyses were not conducted due to the small sample sizes for some priority populations (Supplementary Information Table 3). Imputation was not conducted due to low levels of missingness for zBMI and SEP in both cohorts (Supplementary Information Table 5).

## Results

### Characteristics of the study population

A total of 9417 children and 101740 person-years of follow-up were analysed in our study. Approximately 75% of the children were from an English-speaking household. There was a steady attrition of participants in both cohorts and by the final waves in our analysis 2926 (63.5%) and 1601 (32.1%) participants were still participating in the B and K cohorts, respectively (see Supplementary Information Table 5). Participants at each wave had complete information on BMI ranging from 92.6 to 99.0% in both cohorts, except for wave 8 in the K cohort (52.7%). There were no missing cultural and ethnic data. Mean baseline zBMI, age and SEP values by priority populations, are shown in Table 1.

**Table 1:** Baseline characteristics by cohort.

Variables	English	Middle East & North Africa	East Asia	South & Central Asia	Europe	Africa	Americas	Oceania	Aboriginal & Torres Strait Islander
B Cohort (*n* = 4506)									
N	3459 (76.76%)	125 (2.77%)	237 (5.26%)	88 (1.95%)	278 (6.17%)	31 (0.69%)	41 (0.91%)	72 (1.60%)	175 (3.88%)
zBMI	0.89 (1.04)	1.11 (1.11)	0.83 (1.15)	0.24 (1.23)	0.86 (0.96)	0.61 (1.11)	1.14 (1.02)	1.03 (1.21)	0.86 (1.15)
Age	2.87 (0.01)	2.87 (0.02)	2.89 (0.02)	2.86 (0.02)	2.85 (0.01)	2.85 (0.05)	2.85 (0.04)	2.87 (0.03)	2.87 (0.02)
Male	1747 (50.51%)	54 (43.20%)	125 (52.74%)	62 (70.45%)	140 (50.36%)	12 (38.71%)	21 (51.22%)	45 (62.50%)	92 (52.57%)
Female	1712 (49.49%)	71 (56.80%)	112 (47.26%)	26 (29.55%)	138 (49.64%)	19 (61.29%)	20 (48.78%)	27 (37.50%)	83 (47.43%)
SEP Quintile 1 (Most disadvantaged)	619 (17.95%)	52 (42.28%)	63 (26.81%)	8 (9.09%)	27 (9.71%)	5 (16.13%)	11 (26.83%)	17 (23.94%)	91 (53.53%)
SEP Quintile 2	736 (21.34%)	21 (17.07%)	31 (13.19%)	12 (13.64%)	54 (19.42%)	6 (19.35%)	6 (14.63%)	13 (18.31%)	37 (21.76%)
SEP Quintile 3	713 (20.67%)	18 (14.63%)	33 (14.04%)	16 (18.18%)	55 (19.78%)	3 (9.68%)	4 (9.76%)	14 (19.72%)	25 (14.71%)
SEP Quintile 4	675 (19.5%)	11 (8.94%)	45 (19.15%)	21 (23.86%)	72 (25.90%)	7 (22.58%)	9 (21.95%)	13 (18.31%)	13 (7.65%)
SEP Quintile 5 (Most advantaged)	706 (20.4%)	21 (17.07%)	63 (26.81%)	31 (35.23%)	70 (25.18%)	10 (32.26%)	11 (26.83%)	14 (19.72%)	4 (2.35%)
K Cohort (*n* = 4415)									
N	3330 (74.75%)	98 (2.22%)	267 (6.05%)	108 (2.45%)	329 (7.45%)	47 (1.06%)	53 (1.20%)	63 (1.43%)	150 (3.40%)
zBMI	0.50 (1.03)	0.79 (1.23)	0.28 (1.08)	0.03 (1.27)	0.59 (1.07)	0.12 (0.89)	0.92 (1.10)	1.24 (1.36)	0.72 (1.40)
Age	6.86 (0.24)	6.93 (0.29)	6.88 (0.26)	6.91 (0.23)	6.85 (0.24)	6.85 (0.26)	6.85 (0.21)	6.91 (0.24)	6.90 (0.25)
Male	1687 (51.12%)	58 (59.18%)	130 (48.69%)	51 (47.22%)	173 (52.58%)	14 (29.79%)	27 (50.94%)	36 (57.14%)	72 (48.00%)
Female	1613 (48.88%)	40 (40.82%)	137 (51.31%)	57 (52.78%)	156 (47.42%)	33 (70.21%)	26 (49.06%)	27 (42.86%)	78 (52.00%)
SEP Quintile 1 (Most disadvantaged)	604 (18.35%)	33 (35.11%)	71 (27.00%)	6 (5.56%)	53 (16.11%)	9 (19.15%)	6 (11.54%)	17 (27.42%)	67 (47.18%)
SEP Quintile 2	673 (20.44%)	28 (29.79%)	24 (9.13%)	10 (9.26%)	55 (16.72%)	7 (14.89%)	9 (17.31%)	14 (22.58%)	41 (28.87%)
SEP Quintile 3	698 (21.20%)	15 (15.96%)	41 (15.59%)	26 (24.07%)	65 (19.76%)	8 (17.02%)	14 (26.92%)	12 (19.35%)	22 (15.49%)
SEP Quintile 4	697 (21.17%)	8 (8.51%)	50 (19.01%)	31 (28.70%)	78 (23.71%)	15 (31.91%)	17 (32.69%)	11 (17.74%)	7 (4.93%)
SEP Quintile 5 (Most advantaged)	620 (18.83%)	10 (10.64%)	77 (29.28%)	35 (32.41%)	78 (23.71%)	8 (17.02%)	6 (11.54%)	8 (12.90%)	5 (3.52%)

Data presented as mean and standard deviation or N (%) unless otherwise indicated.

Statistics based on those with complete information on BMI z-score.

*SEP observations were carried forward from Wave 7 to Wave 8 due to missing data.

*B* birth, *K* kindergarten, *BMI* body-mass index, *SEP* socioeconomic position.

### Multilevel models—Cultural and ethnic groups

Figure 1 shows the association between zBMI and each cultural and ethnic group for the three periods of childhood, compared to children who spoke English at home or were born in an English-speaking country. Across all three periods, we found large, positive differences in zBMI for children from Middle East and North Africa, the Americas, and Oceania and lower zBMI in children from the South and Central Asia and African groups.

In early childhood, children from the Middle East and North Africa (0.33 {0.16, 0.49}) and the Americas (0.30 {0.01, 0.59}) were associated with the largest difference in mean zBMI, whereas children in the South & Central Asian group were associated with –0.58 {–0.78, –0.37} lower zBMI, when compared to children from English-speaking households. In middle childhood, the magnitude of higher zBMI was stronger in children from the Oceania (0.59 {0.40, 0.78}), Middle East and North Africa (0.42 {0.28, 0.56}), the Americas (0.41 {0.18, 0.63}) and Aboriginal and Torres Strait Islanders (0.13 {0.01, 0.26}), whereas children in the South and Central Asian (–0.28 {–0.43, –0.12}), East Asian (–0.11 {–0.21}, –0.02 and African (–0.23 {–0.47, 0.01})) groups were associated with lower zBMI. The adolescent period found similar associations as the middle childhood period, although notably there was a much higher magnitude of effect for Aboriginal and Torres Strait Islander children (0.30 {0.14, 0.46}).

### Multilevel models—Socioeconomic position

Figure 2 presents the association between SEP quintiles and zBMI, compared to children in the most advantaged quintile (Quintile 5) for all three periods of childhood. After adjusting for cultural and ethnic group as a confounder, we find zBMI to be socioeconomically patterned, with increasingly larger positive associations for more disadvantaged children. Children in the least advantaged group (Quintile 1) had the largest difference, of 0.10 {0.02, 0.18}, 0.22 {0.17, 0.27} and 0.23 {0.18, 0.29} zBMI in early childhood, middle childhood and adolescence, respectively, whereas those in quintile 4 had a small difference in zBMI when compared to the most advantaged children (0.03 {-0.04, 0.10} in early childhood, 0.04 {0.01, 0.08} in middle childhood and 0.05 {0.01, 0.09} in adolescence).

The coefficients for each multilevel model are presented in Supplementary Information Tables 7, 8.

## Discussion

Australia is a multicultural society with a strong and continuing migration policy from diverse countries which has led to an increase in its cultural and linguistic diversity [6]. There are public health concerns that children from different priority are disproportionately affected by overweight and obesity [2]. To the best of our knowledge, our study is the first to use longitudinal data from 9417 participants to identify differences in zBMI by cultural, ethnic and socioeconomic groups are associated with higher zBMI during three developmental stages of childhood.

Our results revealed a clear effect of disparities in zBMI for children from different priority populations at three important childhood stages. Children from South and Central Asian, East Asian and African households had a consistently lower zBMI, while those from the Middle East and North Africa, Oceania (excluding Australia and New Zealand) and Americas had consistently higher zBMI than the referent (English) group, at all child developmental ages. Children from European households had similar zBMI to the referent English group. Aboriginal and Torres Strait Islander children had lower zBMI in early childhood, but higher in middle childhood and adolescence. Across all three childhood periods, we consistently found a socioeconomic gradient, with increasingly higher zBMI associated with greater socioeconomic disadvantage.

Our findings indicate that unhealthy weight development during childhood may be culturally patterned and distinctly different across priority populations. Action is needed and additional resources are required to invest in targeted strategies to reduce these disparities in overweight and obesity across all priority populations. We have identified at which period of childhood certain groups are at greater risk, which can help identify optimal timing of interventions for certain age groups, such as early childhood, primary school, or adolescence. For example, prevention in an early childhood setting may not be suitable for Aboriginal and Torres Strait Islander children who have among the lowest zBMI of any cultural group at this age. Similarly, preconception, pregnancy and early infancy programs may be appropriate for prevention initiatives for those that have the highest zBMI between age 2 and 5 years. Middle and high school programs would be suitable for children from Aboriginal and Torres Strait Islander, Americas, Middle East and North African households.

Culturally tailored obesity prevention programs have been found to be effective [28] amongst some cultural and ethnic groups, however only a limited number of programs have been developed in Australia [29]. Currently, there is a lack of programs specifically aimed at addressing Aboriginal childhood overweight and obesity [30], with a need for Aboriginal designed and led initiatives to support self-determination and positive health outcomes [31]. Based on our findings, we suggest a need for more healthy growth programs for Aboriginal and Torres Strait Islander children, and children from the Middle East and North Africa, the Americas and Oceania (excluding Australia and New Zealand) households. Policymakers can use our findings to design strengths-based, community led approaches or culturally adapting existing health programs and public health policies to reduce disparities in children’s weight status in these key groups.

Our results are consistent with both cross-sectional and longitudinal studies in Australia that compared to children from English-speaking backgrounds, children of Middle Eastern and North African, Oceanian and Aboriginal and Torres Strait Islanders had higher prevalence of overweight and obesity whereas children from Asian backgrounds had lower overweight and obesity prevalence [3, 5, 12, 14, 32, 33]. We did not find evidence of higher zBMI in children from a European background, however the existing evidence is mixed, with studies reporting higher [5, 12, 13] and lower [32] odds of overweight and obesity, when compared to children from English-speaking backgrounds.

The strength of this study was the use of a large longitudinal dataset of over 9000 children in Australia with over 100000 person-years of follow-up through childhood. We identified which priority populations are at risk at three important stages of childhood, which provides valuable information for policymakers deciding how to culturally tailor a prevention program and when to intervene in these communities.

Our study has some limitations. Firstly, LSAC did not collect data on self-reported ethnicity or ancestry and our classification of children into nine separate cultural and ethnic groups was conducted using a combination of regions of birth and languages spoken at home using the best available data as recommended [34]. There is potential for children to be misclassified, although 98% of children were classified using two simple decision rules (see Appendix Supplementary Fig. 2). Due to the small sample sizes of some groups, (i.e., Africa and the Americas), we were unable to separate these into further categories to better reflect the diversity of cultural backgrounds of some priority groups in the analysis, nor were we able to stratify our analyses by sex. Finally, our analysis did not use survey weights and although we have a large and diverse sample, our findings may not be representative of the child and adolescent Australian population.

## Conclusion

The prevalence of overweight and obesity is culturally patterned, and disparities exist among children from different priority populations in Australia. Understanding which priority populations are at higher risk of excess weight at different ages is important to allocate additional resources in designing and rolling out culturally tailored healthy weight programs to reduce these disparities. Our findings suggest the use of culturally tailored interventions across pre-primary, primary school and adolescence are needed to reduce the disparities in overweight and obesity amongst children from different priority populations.

### Supplementary information

Supplementary Information

## Data Availability

The Longitudinal Study of Australian Children data are available from the Longitudinal Studies Dataverse website (https://dataverse.ada.edu.au/dataverse/lsac) for those who meet the criteria for access to de-identified LSAC data.

## Code availability

All analyses were conducted in STATA version 17.0 (StataCorp, College Station, TX, USA) and statistical code used to generate the results can be accessed upon request.

## References

[1] NCD Risk Factor Collaboration (NCD-RisC). Worldwide trends in body-mass index, underweight, overweight, and obesity from 1975 to 2016: a pooled analysis of 2416 population-based measurement studies in 128·9 million children, adolescents, and adults. Lancet. 2017;390:2627–42. 10.1016/S0140-6736(17)32129-3 PMC5735219 29029897

[2] Australian Institute of Health and Welfare 2020. Australia’s children. Cat. no. CWS 69. Canberra: AIHW.

[3] O’Dea JA, Dibley MJ (2014). Prevalence of obesity, overweight and thinness in Australian children and adolescents by socioeconomic status and ethnic/cultural group in 2006 and 2012. Int J Public Health.

[4] Commonwealth of Australia 2022. The National Obesity Strategy 2022–2032. Health Ministers Meeting.

[5] Hardy LL, Jin K, Mihrshahi S, Ding D (2019). Trends in overweight, obesity, and waist-to-height ratio among Australian children from linguistically diverse backgrounds, 1997 to 2015. Int J Obes (Lond).

[6] Australian Bureau of Statistics. Cultural diversity: Census [Internet]. Canberra: ABS; 2021. Available from: https://www.abs.gov.au/statistics/people/people-and-communities/cultural-diversity-census/latest-release.

[7] Australian Bureau of Statistics. National, state and territory population [Internet]. Canberra: ABS; 2022 June. Available from: https://www.abs.gov.au/statistics/people/population/national-state-and-territory-population/latest-release.

[8] Popkin BM, Adair LS, Ng SW (2012). Global nutrition transition and the pandemic of obesity in developing countries. Nutr Rev.

[9] Ahmed S, Uddin R, Ziviani J, Gomersall S, Khan A (2022). Lifestyle behaviours of immigrant and Australian children: evidence from a nationally representative sample. Sports Med Health Sci.

[10] Zulfiqar T, Strazdins L, Dinh H, Banwell C, D’Este C (2019). Drivers of overweight/obesity in 4-11 year old children of australians and immigrants; evidence from growing up in Australia. J Immigr Minor Health.

[11] Delavari M, Sønderlund AL, Swinburn B, Mellor D, Renzaho A (2013). Acculturation and obesity among migrant populations in high income countries-a systematic review. BMC Public Health.

[12] Achat HM, Stubbs JM (2014). Socio-economic and ethnic differences in the prevalence of overweight and obesity among school children. J Paediatr Child Health.

[13] Waters E, Ashbolt R, Gibbs L, Booth M, Magarey A, Gold L (2008). Double disadvantage: the influence of ethnicity over socioeconomic position on childhood overweight and obesity: findings from an inner urban population of primary school children. Int J Pediatr Obes.

[14] Australian Bureau of Statistics. National Aboriginal and Torres Strait Islander Health Survey [Internet]. Canberra: ABS; 2018-19. Available from: https://www.abs.gov.au/statistics/people/aboriginal-and-torres-strait-islander-peoples/national-aboriginal-and-torres-strait-islander-health-survey/latest-release.

[15] O’Dea JA, Dibley MJ (2010). Obesity increase among low SES Australian schoolchildren between 2000 and 2006: time for preventive interventions to target children from low income schools?. Int J Public Health.

[16] Hayes AJ, Carrello JP, Kelly PJ, Killedar A, Baur LA (2021). Looking backwards and forwards: tracking and persistence of weight status between early childhood and adolescence. Int J Obes (Lond).

[17] World Health Organization. Report of the commission on ending childhood obesity 2016. ISBN: 9789241510066.

[18] Frohlich KL, Potvin L (2008). Transcending the known in public health practice: the inequality paradox: the population approach and vulnerable populations. Am J Public Health.

[19] Mohal J, Lansangan, C, Gasser, C, Howell, L, Duffy, J, Renda, J et al. Growing up in Australia: the longitudinal study of Australian children—data user guide, Release 9.0C2, June 2022 Melbourne: Australian Institute of Family Studies; 2022.

[20] World Health Organization. Child growth standards. Geneva, Switzerland 2007.

[21] de Onis M, Onyango AW, Borghi E, Siyam A, Nishida C, Siekmann J (2007). Development of a WHO growth reference for school-aged children and adolescents. Bull World Health Organ.

[22] Australian Bureau of Statistics. Standard Australian Classification of Countries (SACC) [Internet]. Canberra: ABS; 2016. Available from: https://www.abs.gov.au/statistics/classifications/standard-australian-classification-countries-sacc/latest-release.

[23] Australian Bureau of Statistics. Australian Standard Classification of Languages (ASCL) [Internet]. Canberra: ABS; 2016. Available from: https://www.abs.gov.au/statistics/classifications/australian-standard-classification-languages-ascl/latest-release.

[24] Australian Bureau of Statistics. Standards for Statistics on Cultural and Language Diversity [Internet]. Canberra: ABS; 2022. Available from: https://www.abs.gov.au/statistics/standards/standards-statistics-cultural-and-language-diversity/latest-release.

[25] Baker K, Sipthorp M, Edwards B A Longitudinal Measure of Socioeconomic Position in LSAC Melbourne: Australian Institute of Family Studies; 2017.

[26] Killedar A, Lung T, Hayes A (2022). Investigating socioeconomic inequalities in BMI growth rates during childhood and adolescence. Obes Sci Pract.

[27] Matza LS, Patrick DL, Riley AW, Alexander JJ, Rajmil L, Pleil AM (2013). Pediatric patient-reported outcome instruments for research to support medical product labeling: report of the ISPOR PRO good research practices for the assessment of children and adolescents task force. Value Health.

[28] Renzaho AM, Mellor D, Boulton K, Swinburn B (2010). Effectiveness of prevention programmes for obesity and chronic diseases among immigrants to developed countries - a systematic review. Public Health Nutr.

[29] Marshall S, Taki S, Laird Y, Love P, Wen LM, Rissel C (2022). Cultural adaptations of obesity-related behavioral prevention interventions in early childhood: A systematic review. Obes Rev.

[30] Browne J, Hayes R, Gleeson D (2014). Aboriginal health policy: is nutrition the ‘gap’ in ‘Closing the Gap’?. Aust N Z J Public Health.

[31] Sherriff SL, Baur LA, Lambert MG, Dickson ML, Eades SJ, Muthayya S (2019). Aboriginal childhood overweight and obesity: the need for Aboriginal designed and led initiatives. Public Health Res Pract.

[32] Hartono S, Cochrane T, Niyonsenga T, Kinfu Y (2021). A longitudinal analysis of the effect of maternal region-of-birth on transitions in children’s bodyweight status from early childhood to late adolescence in Australia: a population-based cohort study. Prev Med.

[33] Sjöholm P, Pahkala K, Davison B, Juonala M, Singh G (2020). Socioeconomic status, remoteness and tracking of nutritional status from childhood to adulthood in an Australian aboriginal birth cohort: the ABC study. BMJ Open.

[34] Pham TTL, Berecki-Gisolf J, Clapperton A, O’Brien KS, Liu S, Gibson K (2021). Definitions of culturally and linguistically diverse (CALD): a literature review of epidemiological research in Australia. Int J Environ Res Public Health.

