# Supplementary material for: Differences in weight status among Australian children and adolescents from priority populations: a longitudinal study
Source: Int J Obes (Lond). 2024 Feb 2;48(5):702–8. doi: 10.1038/s41366-024-01471-0 (PMC11058044; doi:10.1038/s41366-024-01471-0)
Supplement: Supplementary file 1 — Supplementary Information [file 41366_2024_1471_MOESM1_ESM.docx]

**Supplementary Information**

Supplementary Table 1 – Classifications of children in cultural and ethnic groups based on language spoken at home and country of birth

| Cultural and ethnic group | Country list | Language list |
| --- | --- | --- |
| English | Australia, United Kingdom, England, Scotland, Wales, Northern Ireland, Ireland, New Zealand, Canada, USA, South Africa | English, Auslan |
| Middle East & North Africa | Algeria, Egypt, Libya, Morocco, Sudan, Bahrain, Gaza Strip and West Bank, Iran, Iraq, Israel, Jordan, Kuwait, Lebanon, Qatar, Saudi Arabia, Syria, Turkey, United Arab Emirates | Kurdish, Dari, Iranic not elsewhere classified, Arabic, Assyrian including Aramaic, Hebrew, Middle Eastern Semitic Languages not elsewhere classified, Turkish |
| East Asia | Myanmar, Cambodia, Laos, Thailand, Vietnam, Brunei Darussalam, Indonesia, Malaysia, Philippines, Singapore, Timor-Leste, China (excludes SARs and Taiwan), Hong Kong (SAR of China), Macau (SAR of China), Taiwan, Japan, South Korea | Burmese, Burmese and related languages not elsewhere classified, Hmong-Miean, Khmer, Vietnamese, Mon-Khmer, Lao, Thai, Cebuano, Ilokano, Indonesian, Malay, Tetum, Tagalog, Other Southeast Asian languages, Cantonese, Hakka, Hokkien, Teochew, Mandarin, Chinese not elsewhere classified, Japanese, Korean, |
| South and Central Asia | Bangladesh, India, Maldives, Nepal, Pakistan, Sri Lanka, Afghanistan, Armenia, Azerbaijan, Georgia, Kazakhstan, Kyrgyzstan, Tajikistan, Turkmenistan, Uzbekistan | Kannada, Malayalam, Tamil, Telegu, Bengali, Gujurati, Hindi, Konkani, Marathi, Nepali, Punjabi, Sindhi, Sinhalese, Urdu, Indo-Aryan not elsewhere classified, Other Southern Asian langauges |
| Europe | Austria, Belgium, France, Germany, Luxembourg, Netherlands, Switzerland, Denmark, Finland, Norway, Sweden, Gibraltar, Italy, Malta, Portugal, Spain, Albania, Bosnia and Herzegovina, Croatia, Cyprus, North Macedonia, Greece, Moldova, Romania, Slovenia, Serbia, Czechia, Estonia, Hungary, Lithuania, Poland, Russia Federation, Slovakia, Ukraine | Danish, Norwegian, Swedish, Finnish, French, Greek, Portuguese, Spanish, Italian, Maltese, Latvian, Hungarian, Lithuanian, Russian, Ukrainian, Bosnian, Croatian, Slovene, Serbian, Polish, Slovak, Romanian, Albanian, Other Eastern European languages not elsewhere classified |
| Africa | Ghana, Liberia, Nigeria, Sierra Leone, Angola, Eritrea, Ethiopia, Kenya, Malawi, Mauritius, Namibia, Seychelles, Somalia, Eswatini, Tanzania, Uganda, Zambia, Zimbabwe, Southern and Eastern Africa, not elsewhere classified | Acholi, Mauritian Creole, Oromo, Somali, Swahili, Amharic, Tigre, African languages not elsewhere classified, French |
| Americas | South America, no further description, Argentina, Bolivia, Brazil, Chile, Colombia, Ecuador, Guyana, Peru, Uruguay, Venezuela, Costa Rica, El Salvador, Honduras, Mexico, Nicaragua, Bahamas, Caribbean, no further description, Jamaica, Trinidad and Tobago | Spanish, Portuguese, English |
| Oceania | New Caledonia, Papua New Guinea, Solomon Islands, Melanesia/Polynesia not further described, Cook Islands, Fiji, Niue, Samoa, Samoa, American, Tonga | Fijian, Maori Cook Island, Maori New Zealand, Motu, Niue, Samoan, Tongan, Pacific Austronesian languages not elsewhere classified, Oceanian Pidgins and Creoles not elsewhere classified Kiwai |
| Aboriginal and Torres Strait Islander | Variable specified in LSAC | Variable specified in LSAC |

*Lack of languages listed by participants in the study

Supplementary Figure 1 – Cultural and ethnic groups specified by countries (Aboriginal and Torres Strait Islander excluded)


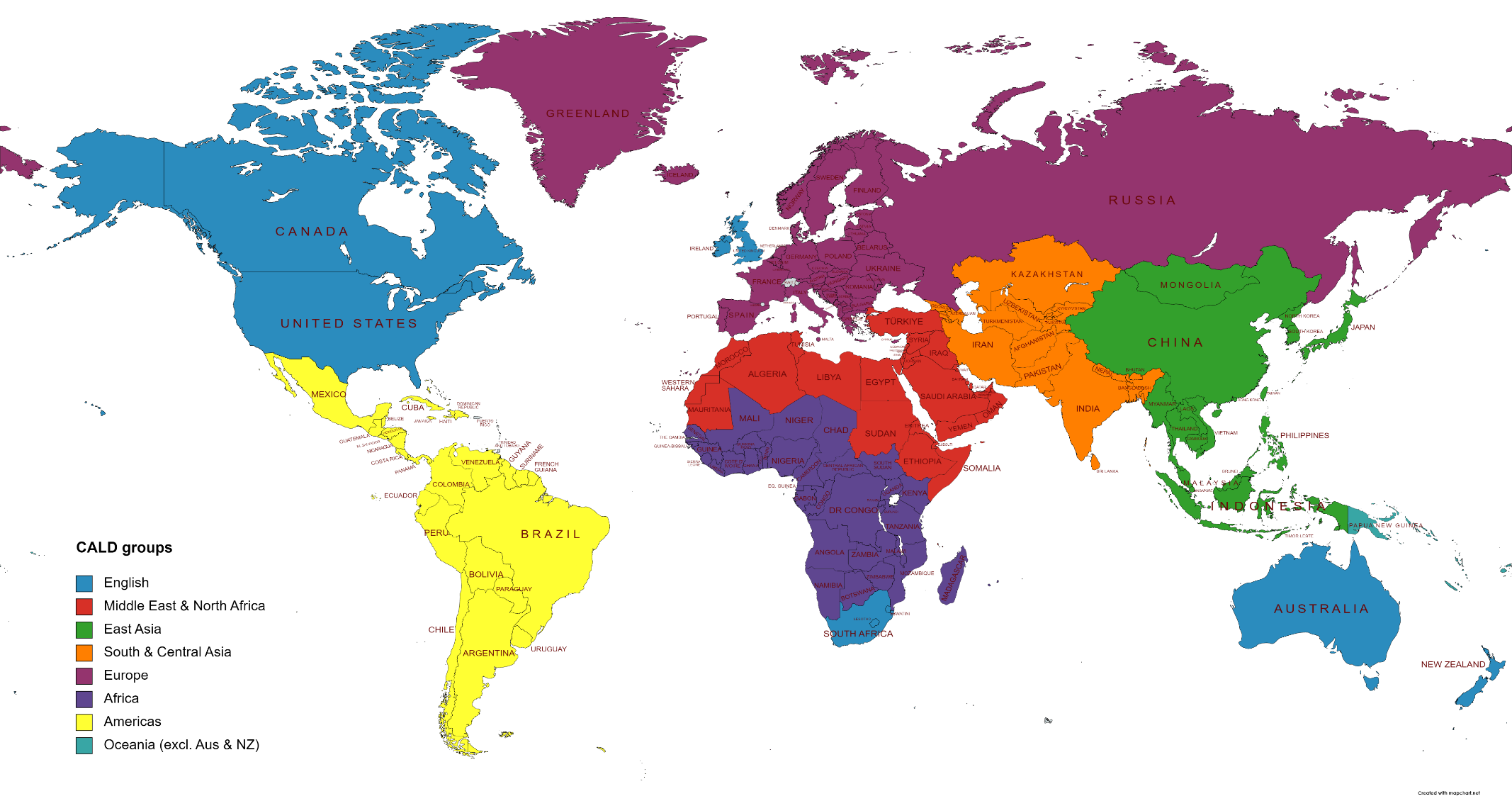


*Aboriginal and Torres Strait Islander not included

Supplementary Table 2 - How children were classified into cultural and ethnic groups

At baseline, children were classified using a combination of 6 variables collected in the Longitudinal Study of Australian Children (LSAC), with the exception of Aboriginal and Torres Strait Islanders as they were defined using a separate variable in LSAC on their Aboriginal and Torres Strait Islander status. For each variable, they were assigned to a priority population group based on their country of birth and language spoken at home using the table above.

| **Country of birth** | **Main language spoken at home** |
| --- | --- |
| 1. In which country was the Study Child born? | 4. Does Study Child speak a language other than English at home? |
| 2. In which country was Parent 1 born? | 5. Does Parent 1 speak a language other than English at home? |
| 3. In which country was Parent 2 born? | 6. Does Parent 2 speak a language other than English at home? |

A series of distinct decision rules were made to classify children to different priority populations based on family’s responses to the six variables. These were conducted sequentially to account for the different combinations of responses from participants.

Supplementary Figure 2 - How children were assigned to different cultural and ethnic groups using the variables ‘country of birth’ and ‘main language spoken at home’ collected in the Longitudinal Study of Australian Children for study child, parent 1 and parent 2


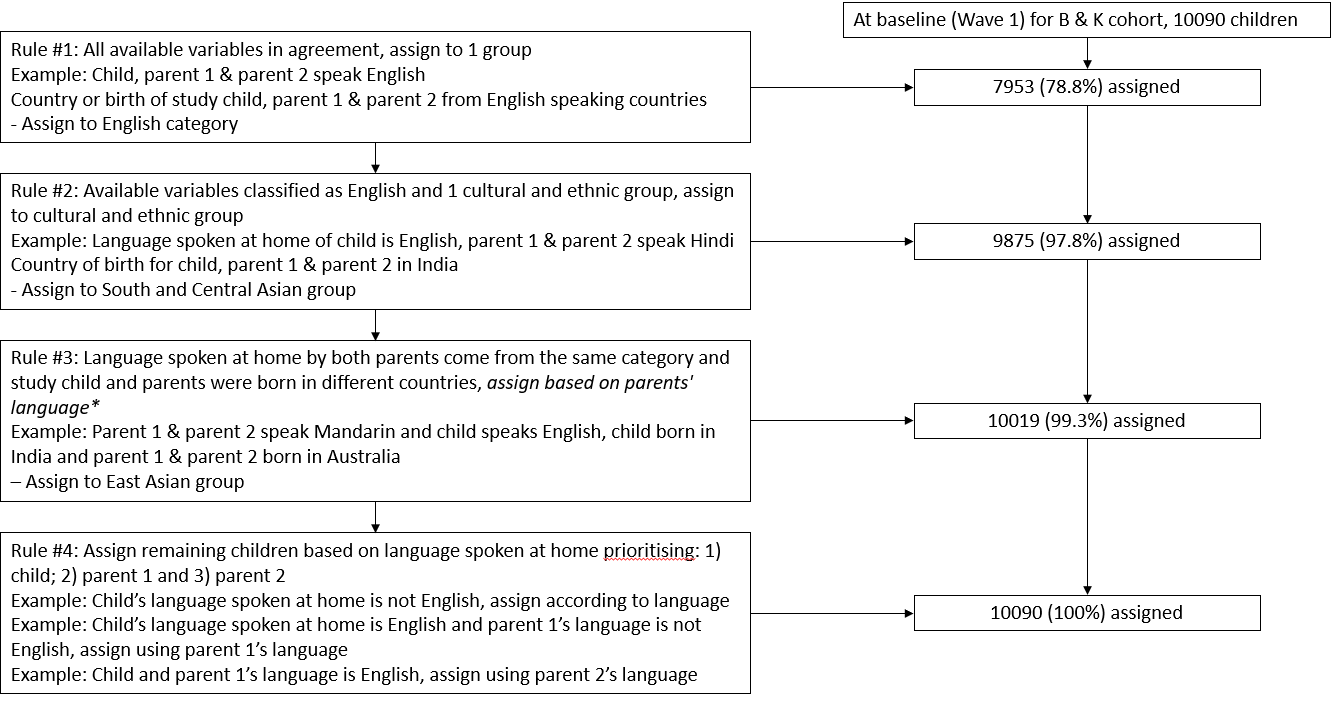


*Exceptions – to account for European colonization of the Americas and Africa, those who spoke a European language (Spanish/Portuguese) and were born in countries from the Americas were assigned to the Americas category, and those who spoke French and were born in African countries were assigned to the African category

Supplementary Table 3 - Sample size of priority populations by wave, cohort and sex

| B cohort | | | | | | | | | | | | | | |
| --- | --- | --- | --- | --- | --- | --- | --- | --- | --- | --- | --- | --- | --- | --- |
| Age | 2-3 years | | 4-5 years | | 6-7 years | | 8-9 years | | 10-11 years | | 12-13 years | | 14-15 years | |
| Wave | 2 | | 3 | | 4 | | 5 | | 6 | | 7 | | 8 | |
| Sex | Male | Female | Male | Female | Male | Female | Male | Female | Male | Female | Male | Female | Male | Female |
| English | 1747 (76.0%) | 1712 (77.5%) | 1706 (77.4%) | 1658 (78.7%) | 1658 (77.3%) | 1594 (78.8%) | 1610 (78.8%) | 1556 (79.9%) | 1430 (78.4%) | 1391 (80.0%) | 1286 (78.4%) | 1238 (81.0%) | 1194 (78.4%) | 1129 (80.5%) |
| Middle East & North Africa | 54 (2.4%) | 71 (3.2%) | 52 (2.4%) | 66 (3.1%) | 52 (2.4%) | 67 (3.3%) | 27 (1.3%) | 29 (1.5%) | 36 (2.0%) | 52 (3.0%) | 34 (2.1%) | 37 (2.4%) | 34 (2.2%) | 37 (2.6%) |
| East Asian | 125 5.4%) | 112 5.1%) | 109 4.9%) | 97 (4.6%) | 109 5.1%) | 94 (4.7%) | 95 (4.7%) | 87 (4.5%) | 93 (5.1%) | 83 (4.8%) | 81 (4.9%) | 73 (4.8%) | 78 (5.1%) | 69 (4.9%) |
| South & Central Asia | 62 (2.7%) | 26 (1.2%) | 56 (2.5%) | 25 (1.2%) | 54 (2.5%) | 20 (1.0%) | 41 (2.0%) | 21 (1.1%) | 49 (2.7%) | 20 (1.2%) | 44 (2.7%) | 13 (0.9%) | 43 (2.8%) | 16 (1.1%) |
| Europe | 140 (6.1%) | 138 (6.3%) | 135 (6.1%) | 132 (6.3%) | 131 (6.1%) | 129 (6.4%) | 97 (4.8%) | 101 (5.2%) | 116 (6.4%) | 102 (5.9%) | 104 (6.3%) | 94 (6.2%) | 93 (6.1%) | 86 (6.1%) |
| Africa | 12 (0.5%) | 19 (0.9%) | 10 (0.5%) | 17 (0.8%) | 8 (0.4%) | 16 (0.8%) | 26 (1.3%) | 31 (1.6%) | 6 (0.3%) | 14 (0.8%) | 4  (0.2%) | 11 (0.7%) | 4 (0.3%) | 10 (0.7%) |
| Americas | 21 (0.9%) | 20 (0.9%) | 21 (1.0%) | 18 (0.9%) | 18 (0.8%) | 18 (0.9%) | 45 (2.2%) | 34 (1.8%) | 16 (0.9%) | 14 (0.8%) | 16 (1.0%) | 13 (0.9%) | 17 (1.1%) | 11 (0.8%) |
| Oceania | 45 (2.0%) | 27 (1.2%) | 41 (1.9%) | 23 (1.1%) | 39 (1.8%) | 19 (0.9%) | 32 (1.6%) | 24 (1.2%) | 27 (1.5%) | 19 (1.1%) | 28 (1.7%) | 14 (0.9%) | 24 (1.6%) | 13 (0.9%) |
| Aboriginal & Torres Strait Islander | 92 (4.0%) | 83 (3.8%) | 75 (3.4%) | 70 (3.3%) | 76 (3.5%) | 66 (3.3%) | 69 (3.4%) | 64 (3.3%) | 50 (2.7%) | 44 (2.5%) | 43 (2.6%) | 36 (2.4%) | 36 (2.4%) | 32 (2.3%) |
| K cohort | | | | | | | | | | | | | | |
| Age | 6-7 years | | 8-9 years | | 10-11 years | | 12-13 years | | 14-15 years | | 16-17 years | | 18 years | |
| Wave | 2 | | 3 | | 4 | | 5 | | 6 | | 7 | | 8 | |
| Sex | Male | Female | Male | Female | Male | Female | Male | Female | Male | Female | Male | Female | Male | Female |
| English | 1687 (75.0%) | 1613 (74.4%) | 1666 (76.0%) | 1586 (75.7%) | 1575 (76.5%) | 1478 (76.0%) | 1499 (77.0%) | 1413 (76.2%) | 1315 (77.8%) | 1220 (77.3%) | 1153 (78.0%) | 1062 (76.9%) | 583 (76.6%) | 551 (74.1%) |
| Middle East & North Africa | 58 (2.6%) | 40 (1.9%) | 52 (2.4%) | 30 (1.4%) | 48 (2.3%) | 24 (1.2%) | 39 (2.0%) | 21 (1.1%) | 32 (1.9%) | 14 (0.9%) | 26 (1.8%) | 15 (1.1%) | 13 (1.7%) | 10 (1.3%) |
| East Asian | 130 (5.8%) | 137 (6.3%) | 128 (5.8%) | 131 (6.3%) | 115 (5.6%) | 120 (6.2%) | 114 (5.9%) | 117 (6.3%) | 98 (5.8%) | 102 (6.5%) | 86 (5.8%) | 91 (6.6%) | 53 (7.0%) | 59 (7.9%) |
| South & Central Asia | 51 (2.3%) | 57 (2.6%) | 49 (2.2%) | 58 (2.8%) | 46 (2.2%) | 51 (2.6%) | 42 (2.2%) | 54 (2.9%) | 44 (2.6%) | 48 (3.0%) | 37 (2.5%) | 36 (2.6%) | 25 (3.3%) | 21 (2.8%) |
| Europe | 173 (7.7%) | 156 (7.2%) | 164 (7.5%) | 148 (7.1%) | 155 (7.5%) | 144 (7.4%) | 144 (7.4%) | 129 (7.0%) | 122 (7.2%) | 104 (6.6%) | 101 (6.8%) | 96 (7.0%) | 57 (7.5%) | 65 (8.7%) |
| Africa | 14 (0.6%) | 33 (1.5%) | 16 (0.7%) | 30 (1.4%) | 14 (0.7%) | 29 (1.5%) | 12 (0.6%) | 27 (1.5%) | 10 (0.6%) | 21 (1.3%) | 8  (0.5%) | 23 (1.7%) | 4 (0.5%) | 12 (1.6%) |
| Americas | 27 (1.2%) | 26 (1.2%) | 25 (1.1%) | 24 (21.2%) | 22 (1.1%) | 23 (1.2%) | 19 (1.0%) | 24 (1.3%) | 14 (0.8%) | 18 (1.1%) | 17 (1.2%) | 17 (1.2%) | 8 (1.1%) | 13 (1.8%) |
| Oceania | 36 (1.6%) | 27 (1.3%) | 35 (1.6%) | 25 (1.2%) | 31 (1.5%) | 24 (1.2%) | 28 (1.4%) | 18 (1.0%) | 22 (1.3%) | 14 (0.9%) | 19 (1.3%) | 12 (0.9%) | 9 (1.2%) | 1 (0.1%) |
| Aboriginal & Torres Strait Islander | 72 (3.2%) | 78 (3.6%) | 58 (2.6%) | 63 (3.0%) | 52 (2.5%) | 52 (2.7%) | 51 (2.6%) | 51 (2.8%) | 34 (2.0%) | 38 (2.4%) | 31 (2.1%) | 30 (2.2%) | 9 (1.2%) | 12 (1.6%) |

Supplementary Table 4 - Mean BMI z-score values by cultural and ethnic groups by age

| Age | 2-3 yrs | 4-5 yrs | 6-7 yrs | 8-9 yrs | 10-11 yrs | 12-13 yrs | 14-15 yrs | 16-17 yrs | 18-19 yrs |
| --- | --- | --- | --- | --- | --- | --- | --- | --- | --- |
| English | 0.89 (1.04) | 0.65 (1.04) | 0.51 (1.05) | 0.56 (1.13) | 0.52 (1.17) | 0.46 (1.16) | 0.46 (1.13) | 0.46 (1.17) | 0.49 (1.25) |
| Middle East & North Africa | 1.11 (1.11) | 1.08 (1.25) | 0.86 (1.26) | 0.97 (1.30) | 1.02 (1.24) | 0.84 (1.21) | 0.79 (1.35) | 0.94 (1.23) | 0.92 (1.39) |
| East Asia | 0.83 (1.15) | 0.56 (1.17) | 0.36 (1.09) | 0.40 (1.20) | 0.47 (1.21) | 0.36 (1.14) | 0.33 (1.09) | 0.27 (1.10) | 0.18 (1.18) |
| South & Central Asian | 0.24 (1.23) | 0.15 (1.22) | 0.15 (1.27) | 0.36 (1.32) | 0.31 (1.32) | 0.19 (1.35) | 0.09 (1.22) | -0.03 (1.21) | 0.06 (1.15) |
| Europe | 0.86 (0.96) | 0.67 (1.04) | 0.58 (1.08) | 0.61 (1.19) | 0.62 (1.22) | 0.55 (1.20) | 0.47 (1.12) | 0.49 (1.18) | 0.56 (1.21) |
| Africa | 0.61 (1.11) | 0.56 (1.21) | 0.22 (1.00) | 0.44 (1.12) | 0.34 (1.24) | 0.19 (1.28) | 0.41 (1.15) | 0.31 (1.01) | 0.06 (1.30) |
| Americas | 1.14 (1.02) | 0.98 (1.00) | 0.85 (1.10) | 1.12 (1.40) | 0.97 (1.25) | 0.88 (1.13) | 0.77 (0.98) | 0.78 (0.99) | 0.85 (1.02) |
| Oceania | 1.03 (1.21) | 0.93 (1.30) | 1.03 (1.38) | 1.14 (1.48) | 1.00 (1.34) | 0.99 (1.47) | 0.89 (1.44) | 1.27 (1.26) | 1.26 (1.00) |
| Aboriginal & Torres Strait Islander | 0.86 (1.15) | 0.46 (1.12) | 0.58 (1.28) | 0.77 (1.36) | 0.72 (1.42) | 0.79 (1.44) | 0.80 (1.38) | 0.98 (1.46) | 0.58 (1.37) |

Supplementary Table 5 - Number of children interviewed at each wave by cohort, attrition rate and those with complete information on body-mass index and priority population

| B Cohort | | | |
| --- | --- | --- | --- |
| Wave | Age of children | Children interviewed | Complete information on BMI |
| 2 | 2-3 | 4606 | 4506 (97.83%) |
| 3 | 4-5 | 4386 | 4311 (98.29%) |
| 4 | 6-7 | 4242 | 4168 (98.26%) |
| 5 | 8-9 | 4085 | 3989 (97.65%) |
| 6 | 10-11 | 3764 | 3562 (94.63%) |
| 7 | 12-13 | 3381 | 3169 (93.73%) |
| 8 | 14-15 | 3127 | 2926 (93.57%) |
| K Cohort | | | |
| 2 | 6-7 | 4464 | 4415 (98.90%) |
| 3 | 8-9 | 4331 | 4288 (99.01%) |
| 4 | 10-11 | 4169 | 4003 (96.02%) |
| 5 | 12-13 | 3956 | 3802 (96.11%) |
| 6 | 14-15 | 3537 | 3270 (92.45%) |
| 7 | 16-17 | 3089 | 2860 (92.59%) |
| 8 | 18-19 | 3037 | 1601 (52.72%) |

Supplementary Table 6 -Missing data for variables used to classify priority populations at baseline

| Variable | B | K |
| --- | --- | --- |
| Country of birth – Study Child | 0 | 0 |
| Country of birth – Parent 1 | 0 | 1 |
| Country of birth – Parent 2 | 478 | 697 |
| Main language spoken at home – Study Child | 3 | 0 |
| Main language spoken at home – Parent 1 | 0 | 0 |
| Main language spoken at home – Parent 2 | 477 | 697 |
| Aboriginal and Torres Strait Islander status | 175 | 150 |

Supplementary Table 7 – Multilevel linear regression models examining cultural and ethnic groups and the association with BMI z-score across three periods of childhood

| Period | Early childhood | Middle childhood | Adolescence |
| --- | --- | --- | --- |
| Sample | n=4715 | n=8992 | n=7348 |
| Age | (2-5 years) | (6-11 years) | (12-18 years) |
| **Variables** | β (95% CI) | β (95% CI) | β (95% CI) |
| Cultural and ethnic groups (referent: English speaking) | | | |
| Middle East & North African | 0.33 (0.16, 0.49)** | 0.42 (0.28, 0.56)** | 0.34 (0.16, 0.53)** |
| East Asian | -0.08 (-0.21, 0.04) | -0.11 (-0.21, -0.02)* | -0.15 (-0.26, -0.03)* |
| South & Central Asian | -0.58 (-0.78, -0.37)** | -0.28 (-0.43, -0.12)** | -0.32 (-0.50, -0.15)** |
| European | 0.00 (-0.11, 0.12) | 0.08 (-0.01, 0.17) | 0.06 (-0.04, 0.16) |
| African | -0.13 (-0.47, 0.21) | -0.23 (-0.47, 0.01) | -0.20 (-0.49, 0.09) |
| Americas | 0.30 (0.01, 0.59)** | 0.41 (0.18, 0.63) ** | 0.40 (0.15, 0.66)** |
| Oceania | 0.21 (-0.02, 0.43) | 0.59 (0.40, 0.78)** | 0.62 (0.39, 0.85)** |
| Aboriginal & Torres Strait Islander | -0.07 (-0.21, 0.07) | 0.13 (0.01, 0.26)* | 0.30 (0.14, 0.46)** |
| Between-children variance | 0.69 (0.65, 0.73) | 1.08 (1.05, 1.11) | 1.13 (1.09, 1.17) |
| Intraclass correlation coefficient | 0.61 | 0.81 | 0.83 |

*p<0.05, **p<0.01.

Supplementary Table 8 – Multilevel linear regression models examining socioeconomic position and the association with BMI z-score across three periods of childhood, adjusting for the confounder of cultural and ethnic group

| Period | Early childhood | Middle childhood | Adolescence |
| --- | --- | --- | --- |
| Sample | n=4704 | n=8968 | n=7304 |
| Age | (2-5 years) | (6-11 years) | (12-18 years) |
| **Variables** | β (95% CI) | β (95% CI) | β (95% CI) |
| Socioeconomic position (referent Quintile 5, most advantaged) | | | |
| SEP Quintile 1 | 0.10 (0.02, 0.18)* | 0.22 (0.17, 0.27)** | 0.23 (0.18, 0.29)** |
| SEP Quintile 2 | 0.09 (0.01, 0.16)* | 0.17 (0.13, 0.22)** | 0.19 (0.13, 0.24)** |
| SEP Quintile 3 | 0.04 (-0.03, 0.12) | 0.10 (0.06, 0.14)** | 0.09 (0.04, 0.14)** |
| SEP Quintile 4 | 0.03 (-0.04, 0.10) | 0.04 (0.01, 0.08)* | 0.05 (0.01, 0.09)* |
| Cultural and ethnic groups (referent: English speaking) | | | |
| Middle East & North African | 0.33 (0.15, 0.50)** | 0.39 (0.25, 0.53)** | 0.30 (0.11, 0.49)** |
| East Asian | -0.07 (-0.19, 0.06) | -0.11 (-0.21, -0.01)* | -0.15 (-0.27, -0.04)** |
| South & Central Asian | -0.56 (-0.76, -0.36)** | -0.24 (-0.39, -0.09)** | -0.29 (-0.46, -0.12)** |
| European | 0.01 (-0.10, 0.13) | 0.10 (0.01, 0.19)* | 0.08 (-0.02, 0.18) |
| African | -0.13 (-0.47, 0.21) | -0.21 (-0.45, 0.03) | -0.17 (-0.46, 0.12) |
| Americas | 0.30 (0.01, 0.59)* | 0.42 (0.20, 0.65) ** | 0.42 (0.17, 0.67)** |
| Oceania | 0.21 (-0.01, 0.44) | 0.57 (0.39, 0.76)** | 0.58 (0.36, 0.81)** |
| Aboriginal & Torres Strait Islander | -0.08 (-0.23, 0.06) | 0.08 (-0.05, 0.20)* | 0.27 (0.10, 0.43)** |
| Between-children variance | 0.69 (0.65, 0.73) | 1.06 (1.03, 1.10) | 1.12 (1.08, 1.16) |
| Intraclass correlation coefficient | 0.61 | 0.81 | 0.83 |

*p<0.05, **p<0.01.

SEP – Socioeconomic position
